# The dilemma of antibiotic susceptibility and clinical decision-making in a multi-drug-resistant *Pseudomonas aeruginosa* bloodstream infection

**DOI:** 10.3389/fphar.2023.1183332

**Published:** 2023-05-30

**Authors:** Long Chen, Xingyi Qu, Jingqian Su, Haijun Yao, Qiang Yuan, Yu Wang, Nanyang Li, Gang Wu, Xiaofen Liu, Jin Hu, Jing Zhang

**Affiliations:** ^1^ Department of Neurosurgery and Neurocritical Care, Huashan Hospital, Fudan University, Shanghai, China; ^2^ Fudan University and Key Laboratory of Clinical Pharmacology of Antibiotics and National Health Commission and National Clinical Research Center for Aging and Medicine, Institute of Antibiotics, Huashan Hospital, Fudan University, Shanghai, China; ^3^ Phase I Unit, Huashan Hospital, Fudan University, Shanghai, China; ^4^ Fujian Key Laboratory of Innate Immune Biology, Biomedical Research Center of South China, College of Life Science, Fujian Normal University, Fuzhou, China

**Keywords:** therapeutic drug monitoring, pharmacokinetics/pharmacodynamics, multidisciplinary treatment, multi-drug-resistant *Pseudomonas aeruginosa*, bloodstream infection

## Abstract

**Objective:** How to choose the appropriate antibiotics and dosage has always been a difficult issue during the treatment of multi-drug-resistant bacterial infections. Our study aims to resolve this difficulty by introducing our multi-disciplinary treatment (MDT) clinical decision-making scheme based on rigorous interpretation of antibiotic susceptibility tests and precise therapeutic drug monitoring (TDM)-guided dosage adjustment.

**Method:** The treatment course of an elderly patient who developed a multi-drug-resistant *Pseudomonas aeruginosa* (MDRPA) bloodstream infection from a brain abscess was presented.

**Results:** In the treatment process, ceftazidime–avibactam (CAZ–AVI) was used empirically for treating the infection and clinical symptoms improved. However, the follow-up bacterial susceptibility test showed that the bacteria were resistant to CAZ–AVI. Considering the low fault tolerance of clinical therapy, the treatment was switched to a 1 mg/kg maintenance dose of susceptible polymyxin B, and TDM showed that the AUC_24h, ss_ of 65.5 mgh/L had been achieved. However, clinical symptoms were not improved after 6 days of treatment. Facing the complicated situation, the cooperation of physicians, clinical pharmacologists, and microbiologists was applied, and the treatment finally succeeded with the pathogen eradicated when polymyxin B dose was increased to 1.4 mg/kg, with the AUC_24h, ss_ of 98.6 mgh/L.

**Conclusion:** MDT collaboration on the premise of scientific and standardized drug management is helpful for the recovery process in patients. The empirical judgment of doctors, the medication recommendations from experts in the field of TDM and pharmacokinetics/pharmacodynamics, and the drug susceptibility results provided by the clinical microbiology laboratory all provide the direction of treatment.

## Introduction

As an opportunistic Gram-negative bacterium, *Pseudomonas aeruginosa* has been listed as one of the critical priority threatened pathogens with an urgent need for new antibiotics by the World Health Organization (WHO) (Botelho et al., 2019), due to its intrinsic (such as *β*-lactam and penem antibiotics), highly acquired, and adaptive resistance (Azam and Khan, 2019). Multi-drug-resistant *Pseudomonas aeruginosa* (MDRPA) is a challenging worldwide problem, and the mortality rates were 43.2%–58.8% (Kang et al., 2003), which is higher than other Gram-negative bloodstream infections, for example, Enterobacteriaceae and Acinetobacter species (Kang et al., 2005; Micek et al., 2011; Thaden et al., 2017). Although ceftazidime–avibactam (CAZ–AVI) has been considered an effective treatment for multi-drug-resistant or extensively resistant (XDR) *Pseudomonas aeruginosa* infections (Timsit et al., 2020; Corbella et al., 2022), *Pseudomonas aeruginosa* has developed resistance to CAZ–AVI, with the resistance rate ranging from 2% to 18.8% according to resistance surveillance data worldwide in recent years (Wang et al., 2020). Polymyxin B, which is used as a last line of defense against Gram-negative bacterial infection, has been used in the clinical treatment of MDRPA (Nang et al., 2021), despite the dose-limiting nephrotoxicity (Zavascki and Nation, 2017).

The antibiotic treatment options for patients with bacterial infections in clinical practice were largely based on antibiotic susceptibility tests (Langford et al., 2021). However, it does not always guarantee good clinical outcomes due to several reasons (Doern and Brecher, 2011), including but not limited to high-virulent bacteria, impaired host immunity, low concentrations of antibiotics distributed to specific infection sites, and drug–drug interactions among multiple antimicrobials. Therefore, whether to trust the antibiotic susceptibility results and how to choose proper antibiotics during treatments have always been controversial issues in clinical practices. Under the paradoxical conditions, more rigorous interpretation and more precise drug management based on the concept of multi-disciplinary treatment (MDT) are expected to assist in clinical decision-making.

Therapeutic drug monitoring (TDM) and pharmacokinetic/pharmacodynamic (PK/PD)-guided TDM are necessarily recommended to help individualized optimization of antibiotics to improve the clinical efficacy and minimize the toxicity (Tsuji et al., 2019). The antibiotic pharmacokinetics in critically ill patients may perform differently; for example, the half-life (T_1/2_) and %T_>MIC_ of the hydrophilic *β*-lactam antibiotics in critically ill patients were unpredictable (Gonçalves-Pereira and Póvoa, 2011), and the exposure of 1.25 mg/kg in critically ill patients (with the weight of 75 kg) may be similar to that of 2 mg/kg in patients with normal renal function (Xie et al., 2020). More appropriate dosing strategies can be obtained from the actual exposure–effect relationship in the clinic with the patients’ PK closely monitored (Tängdén et al., 2017).

The present report describes a case of bloodstream infection caused by MDRPA that faced critical treatment decisions, combined with antibiotic susceptibility tests and TDM to optimize dose adjustment. With the MDT cooperation of physicians, clinical pharmacologists, and microbiologists, the patient achieved clinical cure outcomes.

## Case description

A 69-year-old male patient (165 cm, 70 kg) was admitted to the neurosurgical intensive care unit (NSICU) following 2 weeks of fever and headache. The patient was diagnosed with abscesses by magnetic resonance and computerized tomography. After treatment for encephalopyosis, the patient’s body temperature and inflammatory indicators returned to its normal range. However, on the 16th day after his admission, the patient’s body temperature and laboratory test indicators increased, suggesting the possibility of infection occurrence. The positive blood bacterial culture of *Pseudomonas aeruginosa* was reported, indicating a bloodstream infection which is possibly due to central venous catheterization. The patient’s medical history showed that vancomycin, meropenem, metronidazole, linezolid, and ceftriaxone were administered before bloodstream infection treatment.

CAZ–AVI (2.5 g, 8-hourly) was administered empirically according to the bacterial epidemiology in the local department. The patient’s symptoms gradually improved, and the infection biomarkers of blood routine tests were decreased, with WBC from 14.65×10^9^/L to 8.01×10^9^/L, CRP from 209.09 mg/L to 29.02 mg/L, and PCT from 0.28 ng/mL to 0.17 ng/mL. However, disc agar diffusion tests revealed that the isolated pathogen was resistant to CAZ–AVI (18 mm, resistant: ≤20 mm, sensitive: >20 mm) and susceptible to polymyxin B and amikacin 3 days later. As shown in Table 1, the antibiotic susceptibility results from the microdilution method showed that this infected *Pseudomonas aeruginosa* strain was resistant to CAZ–AVI (MIC was 16/4 mg/L). Minimum inhibitory concentrations (MICs) of other different antibiotics were also measured by using the broth microdilution method and suggested that the bacterium was only susceptible to polymyxin B and amikacin. The bacterial isolate was sent for whole-genome sequencing for further analysis.

**TABLE 1:** Drug susceptible tests of *Pseudomonas aeruginosa* isolated from blood samples.

Antibiotic	AMK	PMB^a^	FEP	CAZ	TZP	SCF	TGC	IPM	MEM	CIP	LEV	CAZ–AVI
MIC (mg/L)	8	1	≥32	≥64	≥128	≥64/32	>64	≥16	≥16	8	≥8	16/4
Interpretation	S	S	R	R	R	R	—	R	R	R	R	R

S, susceptible; R, resistant; MIC, minimum inhibitory concentration; AMK, amikacin; PMB, polymyxin B; FEP, cefepime; CAZ, ceftazidime; TZP, piperacillin–tazobactam; SCF, cefoperazone–sulbactam; TGC, tigecycline; IPM, imipenem; CIP, ciprofloxacin; LEV, levofloxacin; CAZ–AVI, ceftazidime–avibactam.

^a^ The drug sensitivity breaking point of polymyxin B refers to the European Committee on Antimicrobial Susceptibility Testing (EUCAST) 2022, and other drugs’ sensitivity breaking points refer to CLSI.

**FIGURE 1:**
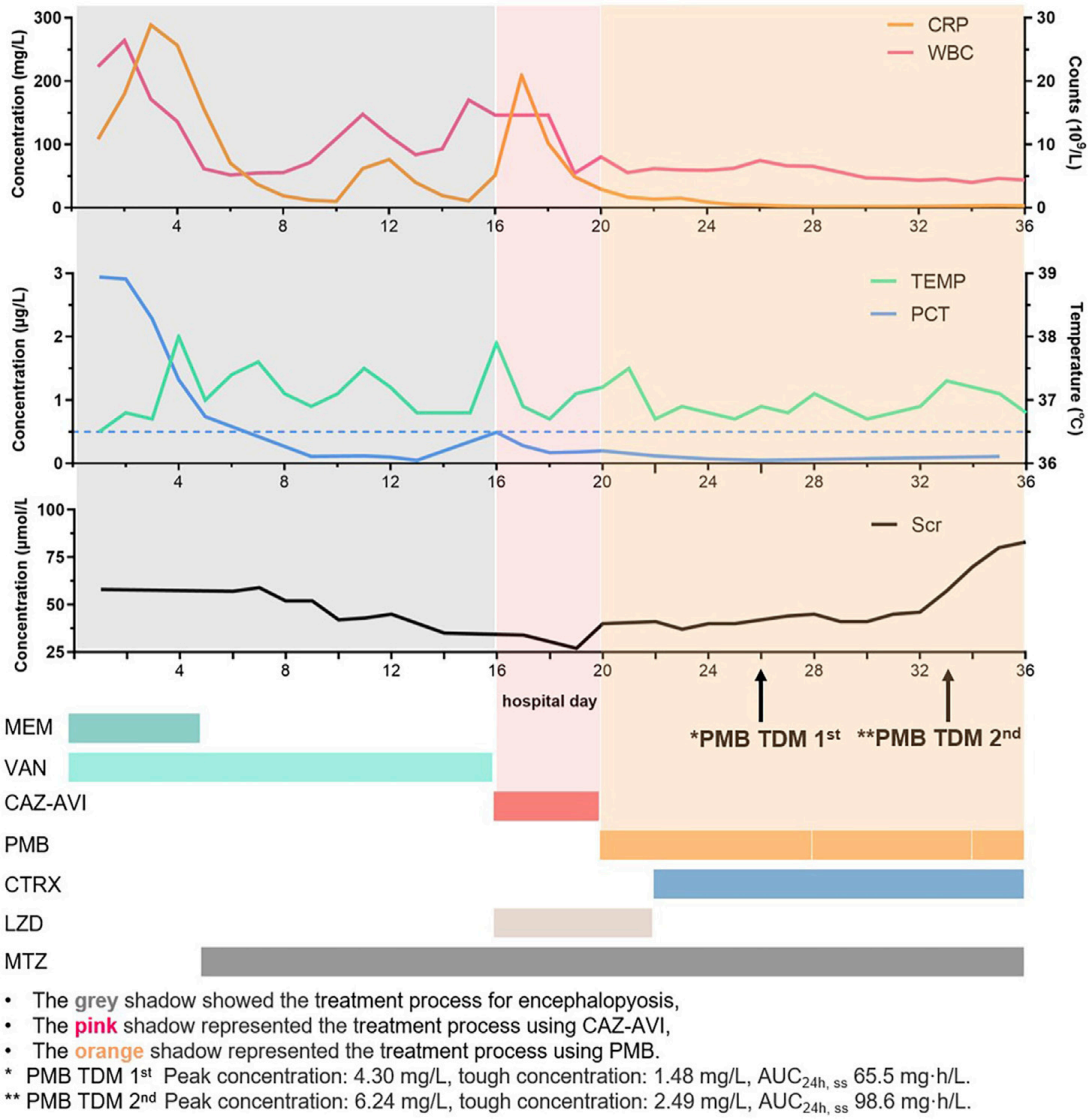
Timeline for the patient's CRP, WBC, PCT, body temperature, creatinine, and drug treatment.

It has been well-recognized that Gram-negative bacteremia can lead to a range of catastrophic clinical outcomes including death (Peña et al., 2012). Particularly, when being infected with carbapenem-resistant *Pseudomonas aeruginosa* (CRPA) or MDRPA, the patients’ 5-day mortality was dramatically increased, which raised great attention (Zhang et al., 2020). Given the low fault tolerance for the clinical therapy, we decided to discontinue CAZ-AVI. Candidate medication regimens include polymyxin B sulfate alone, or its combination with amikacin, both of which were reasonable on the basis of antibiotic susceptibility results (Table 1). However, amikacin was finally abandoned because of its relative high MIC and the upset about nephrotoxicity risk. Polymyxin B has clinical pharmacokinetic advantages (Thaden et al., 2017) and has been reported to eradicate bacteria in bloodstream infection within 3 days (Yu et al., 2022). On hospital day 20, polymyxin B 750,000 units (∼1 mg/kg, 1-h infusion) 12-hourly with 1.5 million units loading dose (∼2 mg/kg, 1-h infusion) was administered, which referred to the International Consensus Guidelines (Tsuji et al., 2019). However, the patient’s body temperature and WBC still showed that the infection had not improved, and the blood cultures were still positive after 6 days of treatment with polymyxin B (Figure 1).

Increasing the dose of polymyxin B needed to be cautious, since nephrotoxicity was a dose-limiting factor, especially for patient’s that had previously received vancomycin for 16 days (for the treatment of a brain abscess) (Wu et al., 2022). Therefore, a multi-disciplinary discussion among physicians in the ICU and infectious disease units, microbiologists, and clinical pharmacologists was conducted to further evaluate the treatment options on the fifth day of polymyxin B administration. PK/PD-TDM-assisted dosing optimization was applied to optimize the polymyxin B dose. The whole blood samples were obtained from the patient before and 30 min after polymyxin B administration on the sixth day and placed into EDTA anticoagulation tubes. The samples were transported to the analytical lab in wet ice, and the concentrations of polymyxin B were determined by using a validated LC-MS/MS method (Liu et al., 2023). In brief, 100 μL plasma samples were precipitated with acetonitrile, and the supernatant was mixed with 6% formic acid in water and injected into LC-MS/MS. The AB Sciex 4500 MD platform interfaced with Shimadzu (Kyoto, Japan) HPLC was employed for LC-MS/MS analysis. Chromatography was gradient-eluted with 0.2% FA (v/v) in acetonitrile and 0.2% FA (v/v) in water, and electrospray ionization (ESI) positive ion and multiple reaction monitoring (MRM) modes were used (Huang et al., 2022; Zhang et al., 2023). Results showed that the polymyxin B concentrations of the peak and trough at a steady state were 4.30 mg/L and 1.48 mg/L, respectively. The AUC_24h, ss_ was 65.5 mg·h/L, calculated by using the two-concentration method and population PK model for the patient (Lakota et al., 2018). The International Consensus Guidelines for the optimal use of polymyxins recommended that AUC_24h, ss_ should be within the range of 50–100 mg·h/L to maximize the clinical efficacy and minimize nephrotoxicity. The Bayesian feedback combined with the population PK model was employed to predict the optimal dose of polymyxin B, and 1 million units of a 12-hourly dose was expected to result in an AUC_ss,0-24h_ of 79.6 mg·h/L, which could achieve the upper limit of the TDM therapeutic window (Yu et al., 2022). Physicians evaluated the patient’s renal function, and the intravenous dose was increased to 1 million units (∼1.4 mg/kg) according to the simulation. TDM was performed again after five doses of adjustments, with the peak and trough concentration, and AUC_0-24h, ss_ was 6.24 and 2.49 mg/L, and 98.6 mg/L·h, respectively. Blood culture showed that MDRPA was eliminated within 3 days after polymyxin B dosing adjustment, and the patient gradually improved from clinical manifestations. Although the etiology had turned negative on hospital day 35, considering that the course of bloodstream treatment is not complete, polymyxin B was continuously administered. The dose of polymyxin B was reduced to 750,000 units due to elevated creatinine levels after 4 days of 1 million units of treatment. Subsequent follow-ups showed creatinine decreased to the baseline when the patient was discharged.

## Discussion

In this case, we encountered evaluations for the treatment options twice. In the first place, the patient’s symptoms improved after CAZ–AVI was administered empirically for *Pseudomonas aeruginosa*-induced bloodstream infection, whereas the susceptibility results showed resistance to CAZ–AVI. There could be several reasons. First, despite the widely accepted guidance of selecting antibiotics based on susceptibility tests, Gary et al. questioned its equivalent with clinical outcomes in the last decade (Doern and Brecher, 2011). MICs from susceptibility tests were obtained through direct interaction between pathogens and antibiotics, without considering the influence of clinical *in vivo* factors (Mouton et al., 2018). For example, the host immunity status affects multiple key steps such as bacterial colonization, expansion, and potential invasion, thus affecting clinical outcomes (Tacconelli et al., 2019; Herrera et al., 2021). Second, various bacterial loads at infection sites also lead to different antibacterial effects. Kim et al. (2020) found that MICs increased by at least eight folds when the bacterial inoculum increased from 10^5^ CFU/mL to 10^7^ CFU/mL, accompanied by increased treatment difficulties. Last, PK/PD simulations of CAZ–AVI showed that for critically ill patients with a creatinine clearance range of 51–190 mL/min, the dose of 2.5 g CAZ–AVI in 2-h infusion 8-hourly could achieve an optimal pharmacodynamic target (*f*%T>MIC) attainment (PTA) (Stein et al., 2019). When MICs of CAZ–AVI were equal to or lower than 16/4 mg/L, PTA was higher than 95% (Stein et al., 2019). The current patient’s creatinine clearance was ∼150 mL/min within the range, which may explain why patients were clinically effective.

A retrospective study from seven hospitals in China found that 40% (151/374) of CRPA strains contained the *bla*_KPC-2_ gene (Zhu et al., 2021). Recently, a novel KPC variant KPC-113 from a clinical *Pseudomonas aeruginosa* was reported to mediate both CAZ–AVI resistance and carbapenem resistance (Yang et al., 2022a). However, the specific mechanism of *Pseudomonas aeruginosa* to CAZ–AVI-resistance needs to be further investigated. For the pathogen isolated in the present study, whole-genome sequencing revealed that neither *bla*_*KPC-2*_- nor bla_KPC-113_-related genes were identified, but instead class C beta-lactamase PDC-5 (bla_AmpC_) was enharbored, which explained that the MIC of CAZ–AVI (16/4 mg/L) is lower than that of CAZ (≥64 mg/L) (Rodríguez-Martínez et al., 2009). In addition, several multidrug efflux pumps (mdtABK, norm, and emrAB) and resistance-nodulation-cell division efflux transporters were detected, contributing to this pathogen’s resistance mechanism (Wu et al., 2022; Yu et al., 2022; Liu et al., 2023).

In the next place, we were caught in a dilemma when 750,000-unit maintenance doses of polymyxin B had been administered for 6 days but showed no clinical symptom improvement. Both the efficacy and toxicity of polymyxin B are related to the PK/PD index AUC/MIC. For isolates with MIC of 2 mg/L or less, AUC_0-24h, ss_ 50–100 mg·h/L is a favorable range for polymyxin B (Lakota et al., 2018). In this case, the polymyxin B MIC of the isolate was 1 mg/L, and we found it difficult to achieve bacterial eradication when AUC_0-24h, ss_ was close to the lower limit of 50 mg·h/L. Our case showed that a higher PK/PD target may be needed for MDRPA-induced bloodstream infection to achieve better clinical efficacy. However, when AUC_0-24h, ss_ achieved ∼100 mg·h/L, the risk of nephrotoxicity also increased. This was consistent with the research by Yang et al. (2022b), in which AUC_0-24h, ss_ >100 mg/L·h was a significant predictor of AKI in critically ill patients. In addition, Yang et al. reported that the trough concentration of 1.2–2.8 mg/L was another index similar to AUC_0-24h, ss_ of 50–100 mg·h/L (34). The 750,000-unit and 1-million-unit maintenance doses achieved trough concentrations of 1.48 mg/L and 2.49 mg/L, respectively. This is consistent with the observation that bacteria are more readily eradicated with higher trough concentrations (2.49 vs. 1.48 mg/L).

TDM and PK/PD-guided dosage optimization were the most important factors for the polymyxin B treatment adjustment. TDM had been highly recommended for polymyxin B dosing to prevent potential nephrotoxicity and achieve the expected clinical efficacy. Therefore, acceptable polymyxin B sampling time, appropriate transportation conditions, and accurate detection methods are all crucial for the evaluation of individual medication plans of patients (Liu et al., 2023). Additionally, scientific and standardized drug management is the premise of achieving accurate and individualized treatment. The experienced judgment of the doctors, the medication suggestions from the experts in the fields of TDM and PK/PD, and the drug sensitivity results provided by the clinical microbiology laboratory all provided the direction for the treatment (Gatti et al., 2022).

## Data Availability

The original contributions presented in the study are included in the article/Supplementary Material; further inquiries can be directed to the corresponding authors.

## Abbreviations

CRP, C-reactive protein; CTRX, ceftriaxone; CAZ–AVI, ceftazidime–avibactam; MEM, meropenem; MTZ, metronidazole; LZD, linezolid; PCT, procalcitonin; PMB, polymyxin B; TEMP, body temperature; VAN, vancomycin; WBC, white blood cell.
